# Alisporivir Treatment Alleviates Mitochondrial Dysfunction in the Skeletal Muscles of C57BL/6NCrl Mice with High-Fat Diet/Streptozotocin-Induced Diabetes Mellitus

**DOI:** 10.3390/ijms22179524

**Published:** 2021-09-02

**Authors:** Konstantin N. Belosludtsev, Vlada S. Starinets, Eugeny Yu. Talanov, Irina B. Mikheeva, Mikhail V. Dubinin, Natalia V. Belosludtseva

**Affiliations:** 1 Department of Biochemistry, Cell Biology and Microbiology, Mari State University, pl. Lenina 1, 424001 Yoshkar-Ola, Russia; vlastar@list.ru (V.S.S.); dubinin1989@gmail.com (M.V.D.); 2 Laboratory of Mitochondrial Transport, Institute of Theoretical and Experimental Biophysics, Russian Academy of Sciences, Institutskaya 3, 142290 Pushchino, Russia; evg-talanov@yandex.ru (E.Y.T.); mikheirina@yandex.ru (I.B.M.); nata.imagination@gmail.com (N.V.B.)

**Keywords:** mitochondria, diabetes mellitus, alisporivir, mitochondrial dysfunction, lipid peroxidation, mitochondrial permeability transition pore

## Abstract

Diabetes mellitus is a systemic metabolic disorder associated with mitochondrial dysfunction, with mitochondrial permeability transition (MPT) pore opening being recognized as one of its pathogenic mechanisms. Alisporivir has been recently identified as a non-immunosuppressive analogue of the MPT pore blocker cyclosporin A and has broad therapeutic potential. The purpose of the present work was to study the effect of alisporivir (2.5 mg/kg/day i.p.) on the ultrastructure and functions of the skeletal muscle mitochondria of mice with diabetes mellitus induced by a high-fat diet combined with streptozotocin injections. The glucose tolerance tests indicated that alisporivir increased the rate of glucose utilization in diabetic mice. An electron microscopy analysis showed that alisporivir prevented diabetes-induced changes in the ultrastructure and content of the mitochondria in myocytes. In diabetes, the ADP-stimulated respiration, respiratory control, and *ADP*/*O* ratios and the level of ATP synthase in the mitochondria decreased, whereas alisporivir treatment restored these indicators. Alisporivir eliminated diabetes-induced increases in mitochondrial lipid peroxidation products. Diabetic mice showed decreased mRNA levels of *Atp5f1a*, *Ant1*, and *Ppif* and increased levels of *Ant2* in the skeletal muscles. The skeletal muscle mitochondria of diabetic animals were sensitized to the MPT pore opening. Alisporivir normalized the expression level of *Ant2* and mitochondrial susceptibility to the MPT pore opening. In parallel, the levels of *Mfn2* and *Drp1* also returned to control values, suggesting a normalization of mitochondrial dynamics. These findings suggest that the targeting of the MPT pore opening by alisporivir is a therapeutic approach to prevent the development of mitochondrial dysfunction and associated oxidative stress in the skeletal muscles in diabetes.

## 1. Introduction

Diabetes mellitus (DM) is a systemic metabolic disease characterized by the impaired production and secretion of insulin due to the destruction of pancreatic beta cells by autoimmune mechanisms (type I diabetes mellitus) or the failure of the cells of the body to adequately respond to insulin, which is mediated by cellular resistance to insulin action on glucose uptake in various tissues, especially the skeletal muscles and adipose tissue (type II diabetes mellitus). As a result, an increase in the blood glucose level (hyperglycemia) and metabolic dysregulation can occur. These disorders ultimately lead to pathological changes in many organs, including skeletal muscle, as this is the largest insulin-sensitive organ of the body [1,2].

It is generally recognized that defects in the structure and functions of mitochondria are important links in the underlying pathogenesis of diabetes at the intracellular level. In particular, decreased glucose uptake by skeletal muscle cells is accompanied by the disorders of mitochondrial fusion and fission, biogenesis, redox homeostasis, and mitophagy [3,4,5,6]. It is important to note that mitochondria-targeted gene or pharmacological therapies restore the ultrastructural organization and functional characteristics of the organelles, reduce the severity of pathological processes associated with oxidative stress, and lead to an increase in the sensitivity of tissues to insulin in diabetic animals and a decrease in the blood glucose level [6,7,8].

A growing body of evidence suggests that the mitochondrial permeability transition (MPT) pore, a nonselective multiprotein channel complex in the inner and outer mitochondrial membranes that is permeable to molecules with a molecular weight of up to 1500 Da, plays a crucial pathogenic role in the development of mitochondrial dysfunction, and it can be considered as a therapeutic target in mitochondria-related metabolic disorders [6,7,8]. The forming of the MPT pore in mitochondria leads to the collapse of membrane potential, the dysregulation of ion homeostasis, the swelling of organelles up until their destruction, and cell death. The excessive accumulation of Ca^2+^ in the mitochondrial matrix and the overproduction of reactive oxygen species are the main triggers of the MPT pore opening in mitochondria [9]. The adenine nucleotide translocator and the ATP synthase are currently considered to be the main proteins involved in the formation of the pore channel and are possibly in alliance with the voltage-dependent anion channel (VDAC) of the outer mitochondrial membrane [10,11,12,13]. Cyclophilin D is a master regulator of the MPT pore, and the inhibitors of this protein cyclosporin A and alisporivir suppress the pore opening at submicromolar concentrations [12,13,14].

Despite the fact that the MPT pore opening participates in many cellular pathologies, the data on its involvement in the pathology of diabetes mellitus are still highly controversial. A number of studies indicate that the diabetic heart and skeletal muscles exhibit a decrease in mitochondrial susceptibility to the MPT pore opening [8,15,16]. At the same time, the liver mitochondria of diabetic animals are more resistant to pore induction [8,17,18,19]. The pharmacological and genetic modulation of the MPT pore results in different, sometimes completely opposite effects in cell cultures and animal models. In particular, cyclophilin D knockout mice were resistant to high-fat diet-induced glucose intolerance and demonstrated improved glucose uptake by the skeletal myocytes [20]. In contrast, cyclophilin D knockout or inhibition exacerbated diabetic renal injury and did not restore mitochondrial functions in the diabetic kidney [21]. It should also be noted that some anti-diabetic drugs (metformin, thiazolidinediones, etc.) can stimulate the opening of the MPT pore in isolated mitochondria [22,23,24]. The above data suggest that the mechanisms of mitochondrial dysfunction in diabetes mellitus are rather tissue-specific and require their targeted regulation. In this regard, the effect of cyclosporin A as a classical inhibitor of the MPT pore is indicative. On the one hand, cyclosporin A prevents the development of mitochondrial dysfunction and improves metabolic parameters in diabetic animals [25,26,27]. On the other hand, cyclosporin A suppresses mitochondrial biogenesis in the human liver cancer cell line, Hep G2 [28]. It should also be pointed out that cyclosporin A is a well-known immunosuppressive agent, and its effects may not be related to the inhibition of the MPT opening [29]. Therefore, the study of the effect of the MPT pore inhibitors that have no side effects would be more representative to elucidate the role of the MPT pore opening in the development of mitochondrial dysfunction in diabetes mellitus.

The aim of this work was to investigate the antidiabetic potential of alisporivir, a non-immunosuppressive blocker of the MPT pore opening [29,30], and its possible protective effect against mitochondrial damage in the skeletal muscles of C57BL/6NCrl mice with diabetes mellitus induced by a high-fat diet combined with streptozotocin injections.

## 2. Results

### 2.1. Effect of Alisporivir on Somatic and Biochemical Characteristics of Mice

**Figure 1:**
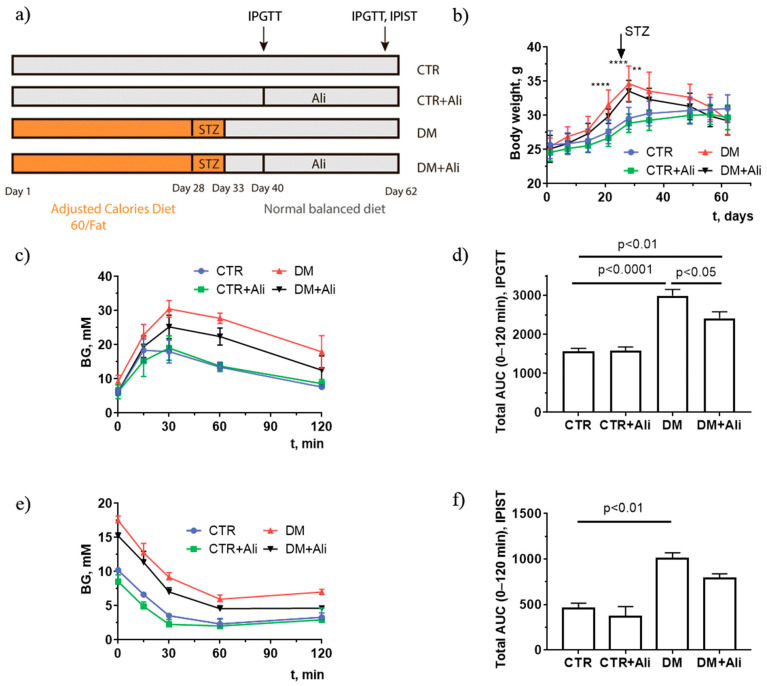
Induction scheme of diabetes mellitus (**a**), body weight gain (**b**), intraperitoneal glucose tolerance test, IPGTT (**c**), and intraperitoneal insulin sensitivity test, IPIST (**e**) in control (CTR), alisporivir-treated control (CTR+Ali), diabetic (DM), and alisporivir-treated diabetic (DM+Ali) mice. The total areas under the curve (AUC) of the IPGTT (**d**) and IPIST (**f**) are shown. The tests were conducted on the 60th day from the beginning of the experiment. ** In subfigure b, the difference between the CTR and DM+Ali groups is significant at *p* < 0.01. **** differences between the CTR and DM groups are significant at *p* < 0.0001. All data are presented as mean ± SEM (*n* = 5). Data in subfigures (**a**,**c**,**d**) are from Belosludtseva et al. *Biology* 2021, *10*, 839, doi: 10.3390/biology10090839.

First, we have determined whether chronic treatment with alisporivir (2.5 mg/kg/day for 21 days) affects the main somatic and biochemical characteristics of diabetic mice. Figure 1 shows the induction scheme of diabetes mellitus, the body weight gain, and the data from an intraperitoneal glucose tolerance test (IPGTT) and an insulin sensitivity test (IPIST) in four experimental groups: (1) non-treated control mice (CTR); (2) alisporivir-treated control mice (CTR+Ali); (3) diabetic mice (DM); and 4) mice with DM treated with alisporivir (DM+Ali). One can see that the administration of alisporivir to the control and diabetic mice had no effect on body weight gain (Figure 1b). At the same time, the results of the IPGTT indicate that alisporivir increased the rate of glucose utilization in diabetic mice (Figure 1c). The data from the IPGTT were also analyzed in terms of the total area under the curve (AUC) between 0 and 120 min (Figure 1d). The AUC of the IPGTT was significantly lower in the alisporivir-treated DM mice than in the DM mice. (Figure 1d). According to the intraperitoneal insulin sensitivity test (IPIST), alisporivir did not significantly alter this parameter in diabetic animals, and the AUC of the IPIST in the DM+Ali group tended to decrease (Figure 1e,f).

### 2.2. Effect of Alisporivir on the Ultrastructure of Skeletal Muscle Mitochondria in Diabetic Mice

Some studies demonstrate that pathological changes in mitochondrial morphology contribute to the development and progression of insulin resistance, obesity, and diabetes mellitus [8,31]. In this regard, in the next part of our work, we studied the effect of alisporivir on the ultrastructure of the mitochondria of the skeletal muscles of the control and diabetic animals.

**Figure 2:**
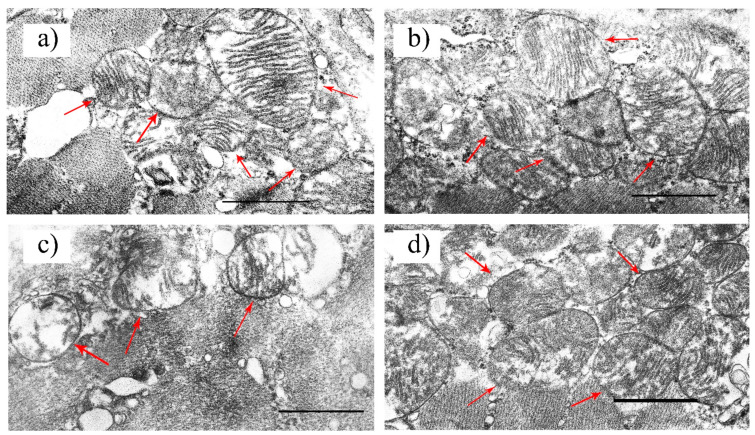
Representative electron micrographs of mouse skeletal muscle mitochondria in the experimental groups: CTR (**a**), CTR + Ali (**b**), DM (**c**), and DM + Ali (**d**). Samples from two skeletal muscles (*M. quadriceps femoris*) were analyzed in each experimental group. The number of the examined fields of view (25 μm^2^) in the groups was 30–50. Red arrows indicate individual mitochondria. Scale bar: 1 μm.

Figure 2 shows representative micrographs of the subsarcolemmal mitochondria of the skeletal muscles of the mice in the experimental groups. In the CTR group, the mitochondria had tightly arrayed cristae surrounded by an intact outer membrane and were bean-shaped or rounded (Figure 2a). The ultrastructural organization of mitochondria in the myocytes of the CTR+Ali group did not differ from that of the CTR group (Figure 2b). Diabetic animals showed spherical mitochondria with a disrupted cristae structure. The formation of the vacuoles and the irregular organization and disorientation of the cristae up until their destruction were observed in the mitochondria. The mitochondria of this group were also characterized by the reduced electron density of the mitochondrial matrix. In some cases, the outer mitochondrial membrane was damaged (Figure 2c). Alisporivir treatment restored the cristae structure and decreased the diabetes-induced vacuolization of the mitochondria. The mitochondrial morphology in the DM+Ali group was similar to that in the CTR and CTR+Ali groups (Figure 2d).

**Figure 3:**
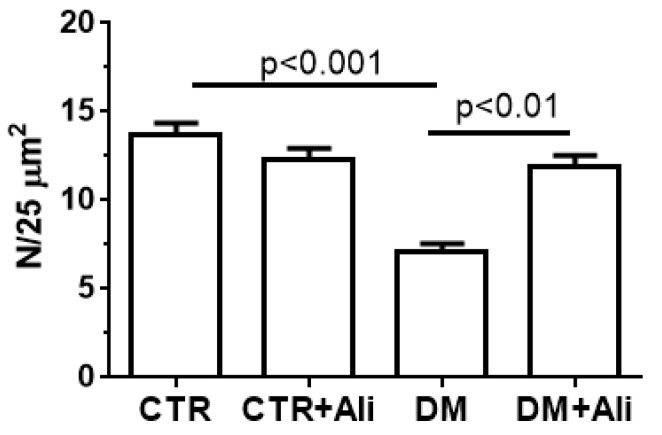
The number of mitochondria per field of view (25 μm^2^). The number of examined fields of view was 30–50 in each group.

The ultrastructural abnormalities of the mitochondria in the skeletal myocytes of the diabetic mice were accompanied by a decrease in the number of organelles. The administration of alisporivir to the diabetic animals (DM+Ali group) resulted in a significant increase in the number of skeletal muscle mitochondria compared to the DM group (Figure 3).

### 2.3. Effect of Alisporivir on DM—Induced Changes in the Functioning of Skeletal Muscle Mitochondria

It was found that diabetes mellitus induces both impairments of the structure of mitochondria and changes in the mitochondrial function in skeletal muscle [8,32]. Here, we evaluated the effect of alisporivir on the main functional parameters of the isolated mitochondria of the skeletal muscles of diabetic animals. Table 1 shows the indicators of the respiration and oxidative phosphorylation of the skeletal muscle mitochondria of the experimental mice. The respiratory substrates glutamate and malate were used to drive complex I-dependent electron transport. The rate of ADP-stimulated mitochondrial respiration (state 3) was significantly decreased (by 23%) in the DM group compared to the CTR one. The diabetic animals demonstrated a decrease in the respiratory control ratio (RCR) and the ADP/O coefficient. The treatment of diabetic mice with alisporivir (DM+Ali group) resulted in a significant increase in the state 3 respiration rate and in the RCR relative to the DM group.

**Figure 4:**
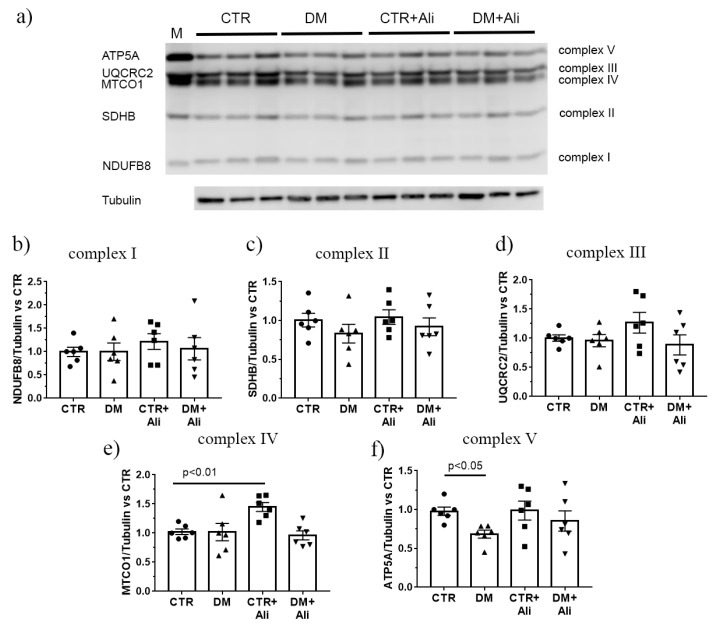
Levels of the proteins of mitochondrial respiratory chain complexes. (**a**) Data from the Western blot analysis. The letter of “M” indicates a positive control (rat heart tissue lysate—mitochondrial extract). Quantification of complex I/tubulin ratio (**b**), complex II/tubulin ratio (**c**), complex III/tubulin ratio (**d**), complex IV/tubulin ratio (**e**), and complex V/tubulin ratio (ATP synthase, **f**). The data are presented as mean ± SEM (*n* = 6).

Then, Western blot analysis was performed to assess the level of the respiratory chain complex subunits in the mitochondria of the four experimental groups. Figure 4 shows that the amount of complexes I–IV in the control and DM groups was the same. Simultaneously, the level of complex V (α-subunit of ATP synthase, ATP5A) was significantly lower in diabetic mice than in the control ones. In the alisporivir-treated diabetic mice, the ATP5A level tended to recover.

**Figure 5:**
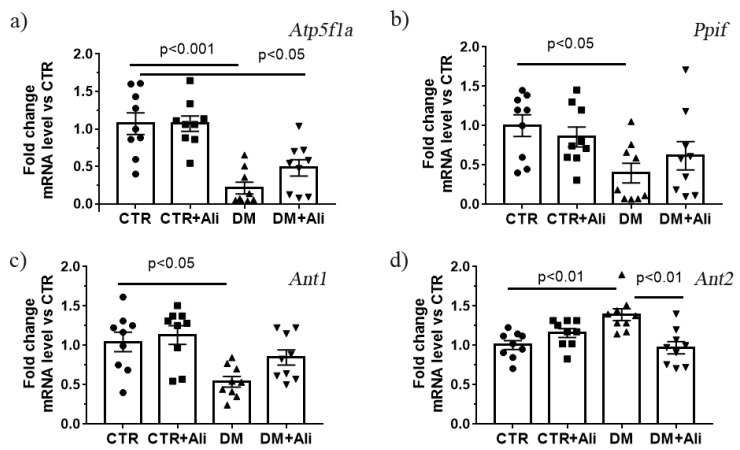
The relative mRNA levels of *Atp5f1a* (**a**), *Ppif* (**b**), *Ant1* (**c**), and *Ant2* (**d**) in the skeletal muscles of experimental animals. The values are given as mean ± SEM (*n* = 9).

It is important to note that a similar pattern was revealed while studying the expression level of the gene encoding the α-subunit of the ATP synthase (Figure 5a). Moreover, the skeletal muscles of the diabetic animals showed decreased expression of the *Ant1* gene encoding the protein responsible for the ADP/ATP antiport in mitochondria (Figure 5c). A decrease in ANT1 can also lead to the suppression of oxidative phosphorylation [33].

**Figure 6:**
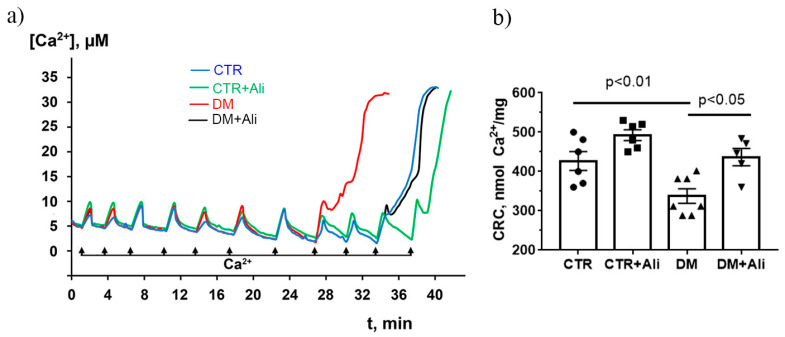
Changes in the external (Ca^2+^) upon the successive addition of Ca^2+^ pulses (10 μM) to the suspension of the skeletal muscle mitochondria of the experimental animals (**a**). Ca^2+^ retention capacity of skeletal muscle mitochondria of experimental animals (**b**). The values are given as means ± SEM (*n* = 6).

Since alisporivir acts as a selective inhibitor of cyclophilin D [30], it is important to evaluate the effect of drug administration on the indicators of the MPT pore opening in mitochondria. As shown in Figure 6, the Ca^2+^ retention capacity of the skeletal muscle mitochondria of diabetic mice decreased 1.25 times compared to that in the control group. This suggests that the skeletal muscle mitochondria of diabetic animals have become less resistant to the MPT pore opening. Alisporivir administration to diabetic animals (DM+Ali group) resulted in a significant increase in this indicator. It should be noted that the treatment of control animals with alisporivir (CTR+Ali group) tended to increase the Ca^2+^ retention capacity as well.

The increase in the Ca^2+^ capacity upon the administration of alisporivir to the diabetic mice may be associated with both the direct action of this compound on mitochondria and its effect on the expression of the proteins that build up the MPT pore, namely ATP synthase, ANTs, and cyclophilin D. As mentioned above, the induction of diabetes mellitus in the experimental mice was accompanied by a decrease in the ATP synthase (both at the level of protein and mRNA). The expression level of ANTs and cyclophilin D is shown in Figs. 5b-d. One can see that diabetic animals exhibited decreased expression of the *Ant1* and *Ppif* genes. The treatment of the diabetic animals with alisporivir did not change the expression level of these genes. At the same time, the DM group revealed an increase in the expression of the gene encoding ANT2 protein. In this case, the treatment of the diabetic animals with alisporivir resulted in a significant decrease in the expression of this gene.

**Figure 7:**
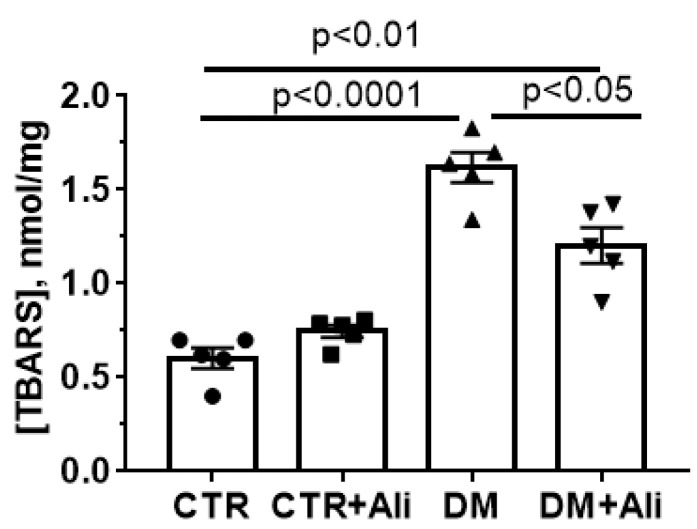
Alisporivir treatment suppresses the DM-induced lipid peroxidation in mouse skeletal muscle mitochondria. Lipid peroxidation was assessed by the level of TBARS in the skeletal muscle mitochondria of the experimental animals. All data are mean ± SEM (*n* = 5).

Oxidative stress is one of the key pathogenic factors that promotes the opening of the MPT pore in mitochondria at the cellular level and contributes to tissue damage [8,9,11,12,13]. Figure 7 shows that diabetic mice demonstrated increased levels of lipid peroxidation products (mainly, malondialdehyde) in skeletal muscle mitochondria, which were quantified by thiobarbituric reactive substance assay. The administration of alisporivir to diabetic animals resulted in the suppression of lipid peroxidation in skeletal muscle mitochondria.

### 2.4. Effect of Alisporivir on Diabetes—Induced Changes in mRNA Levels of Proteins Responsible for Mitochondrial Biogenesis and Dynamics

**Figure 8:**
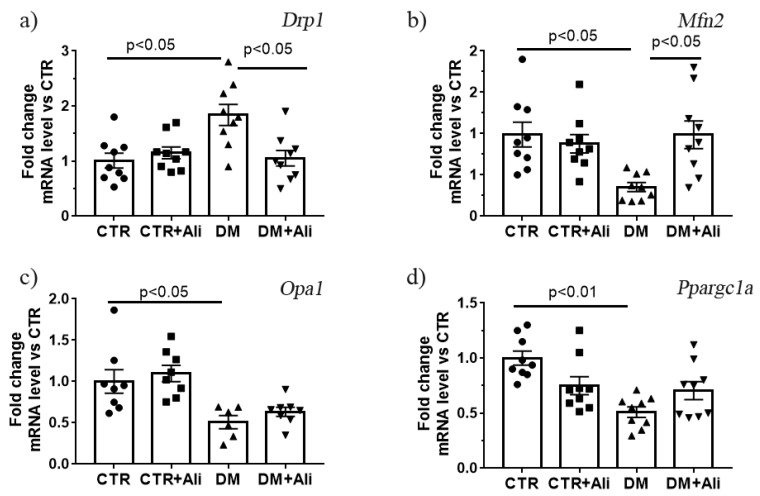
The relative mRNA levels of *Drp1* (**a**), *Mfn2* (**b**), *Opa1* (**c**), and *Ppargc1a* (**d**) in the skeletal muscle of experimental animals. The values are given as mean ± SEM (*n* = 6–9).

Mitochondrial dynamic and biogenesis disorders are the most frequently proposed mechanisms of mitochondrial dysfunction in diabetes mellitus [8]. In the present study, we have determined the changes in the expression of the *Drp1*, *Mfn2*, *Opa1,* and *Ppargc1a* genes, which encode the proteins responsible for mitochondrial fission, fusion, and biogenesis. One can see that the DM group demonstrated an increase in the expression of the gene encoding DRP1 and a decrease in the expression levels of *Mfn2* and *Opa1* (Figure 8). The DM+Ali group showed a decrease in the *Drp1* gene expression and an increase in the *Mfn2* (but not *Opa1*) gene expression levels relative to the DM group. It can be seen that the DM group exhibited a significant decrease in the expression of the *Ppargc1a* gene, which may indicate the suppression of mitochondrial biogenesis upon the development of diabetes mellitus. The administration of alisporivir did not significantly affect the diabetes-induced suppression of biogenesis; however, there was a tendency to an increase in the level of expression of this gene.

## 3. Discussion

The opening of the MPT pore under conditions of cellular stress is currently considered as one of the most important molecular mechanisms leading to mitochondrial dysfunction, cell death, and the consequent development of various pathologies in the vital organs and tissues of humans and animals [12,13]. However, there is no consensus on the role of MPT pore in the pathogenesis of diabetes mellitus and insulin resistance. This may be due to the fact that mitochondrial dysfunction in diabetes mellitus is time and organ specific, which contributes to the development of the disease and its complications in a plethora of ways [8].

The structural components of the MPT pore are still not precisely known. The only protein that is currently established to be a part of the MPT pore is the regulatory protein cyclophilin D [13]. In this regard, studies on the involvement of the MPT pore in diabetes mellitus and other cellular pathologies specifically focus on the pharmacological and genetic modification of this protein. In this work, we have tried to answer the question of whether the novel inhibitor cyclophilin D and the MPT pore opening alisporivir, a non-immunosuppressive analogue of cyclosporin A, is capable of suppressing the development of mitochondrial dysfunction in skeletal muscle in diabetes mellitus.

Our study demonstrates that the induction of diabetes mellitus by a high-fat diet combined with streptozotocin injections is accompanied by a typical pattern of mitochondrial dysfunction in skeletal muscle cells: damage to mitochondrial ultrastructure (Figure 2), a decrease in the number of organelles (Figure 3), suppression of oxidative phosphorylation capacity (Table 1), enhanced lipid peroxidation (Figure 7), and increased susceptibility of mitochondria to MPT pore induction (Figure 6). Targeting the MPT pore opening with alisporivir (2.5 mg/kg/day for 21 days) noticeably restores the morphology and function of mitochondria in skeletal myocytes of diabetic animals. It is also important to note that the administration of alisporivir to diabetic animals normalizes the resistance of skeletal muscle mitochondria to the MPT pore opening to the control level, which may be due to the direct cyclophilin-binding effect of alisporivir.

As shown in Table 1, diabetes mellitus leads to the reduction of oxidative phosphorylation in mouse skeletal muscle mitochondria. A decrease in the state 3 mitochondrial respiration rate in the DM group can be a consequence of a decline in the levels of two key mitochondrial proteins: ATP synthase (assessed by Western blotting and RT-PCR analyses) and ANT1 (demonstrated by mRNA level), the main isoform of the adenine nucleotide translocator protein in the skeletal and cardiac muscles, which mediates ADP/ATP antiport across the inner mitochondrial membrane. The treatment of diabetic mice with alisporivir results in an increase in the rate of ADP-stimulated respiration of the skeletal muscle mitochondria compared to that of DM mice. At the same time, alisporivir does not significantly increase the amount of the ATP synthase α-subunit and the mRNA levels of *Atp5f1a* and *Ant1* in the skeletal muscles of diabetic animals; however, upward trends are observed. One could suggest that the restoration of the oxidative phosphorylation processes is associated with an increase in the activity of the complexes of the mitochondrial respiratory chain. The activity of the respiratory complexes can be upregulated through an increase in the amount of crucial membrane lipids (cardiolipin), electron carriers (as coenzyme Q), or the level of matrix Ca^2+^ in the mitochondria [18,34].

The normalization of the ultrastructure and oxidative phosphorylation of skeletal muscle mitochondria by alisporivir treatment in diabetes mellitus can be attributed to its effect on the signaling systems responsible for mitochondrial biogenesis (PGC1-α) and mitochondrial dynamics (mitofusin 2 and DRP1). Experimental mice with high-fat diet-/streptozotocin-induced diabetes mellitus reveal a decrease in the expression of the *Ppargc1a* and *Mfn2* and *Opa1* genes as well as an increase in the expression of the *Drp1*. This suggests that the induction of diabetes mellitus is accompanied by a remodeling of the mitochondrial network of myocytes, namely a decrease in mitochondrial biogenesis and mitochondrial fusion as well as an increase in mitochondrial fission episodes. The treatment of diabetic animals with alisporivir does not significantly change the level of the *Ppargc1a* gene expression in relation to the level in the DM group. It should be mentioned that the CTR+Ali group of animals shows a tendency towards a decrease in the level of *Ppargc1a* gene expression (by 25%). Previously, it was shown that cyclosporin A reduced the expression of PGC-1a in HepG2 cell culture [23]. It is possible that its non-immunosuppressive analogue alisporivir has a similar effect. On the other hand, the administration of alisporivir to diabetic animals leads to a decrease in the mRNA level of *Drp1* and an increase in *Mtf2* gene expression. This indicates that alisporivir can restore the mitochondrial dynamic processes in the skeletal myocytes of diabetic animals.

The fact that the administration of alisporivir to diabetic animals results in faster glucose utilization during the glucose tolerance test allows us to consider alisporivir as an agent that promotes the absorption of glucose from the blood. Earlier, it was observed that alisporivir failed to improve renal function and suppress the progression of pathology in db/db mice, as assessed by no change in albuminuria, KIM-1 excretion, and glomerulosclerosis [21]. These findings are in contrast to our results. One of the possible explanations for this contradiction is the use of a different alisporivir administration route in the previous works. In our work, this drug was administered intraperitoneally, while Lindblom et al. used the oral route for its administration. It is possible that the oral administration of alisporivir resulted in its degradation in the gastrointestinal tract. On the other hand, the action of alisporivir, similar to the knockout of cyclophilin D [20], may have a tissue-specific effect in the treatment of diabetes mellitus.

The mechanisms of alisporivir-mediated increased glucose utilization in diabetic animals require further research. On the one hand, it has been demonstrated that the muscle-specific genetic deletion of cyclophilin D protected the mice from high-fat diet-induced glucose intolerance and improved the glucose uptake by the skeletal muscle cells. However, no changes were observed in muscle oxidative damage, insulin signaling, or lipotoxic lipid accumulation [20]. Thus, the down-regulation of muscle cyclophilin D could alleviate the pathological changes in diabetes. On the other hand, there is evidence that the knockout of cyclophilin D can lead to defects in various metabolic pathways and to the impairment of whole-body glucose homeostasis in diabetic mice [29,35].

Based on the results of this work, one can conclude that targeting cyclophilin D and the MPT pore by alisporivir improves the ultrastructure and functioning of skeletal muscle mitochondria in diabetes, and the suppression of oxidative stress associated with the MPT pore opening may underlie the protective action of alisporivir against mitochondrial dysfunction in the skeletal muscles in diabetes mellitus.

## 4. Materials and Methods

### 4.1. Animals and the Induction of Diabetes

Six- to seven-week-old male mice of the C57BL/6NCrl line weighing 23–26 g were used in this work. The animals were purchased from the Animal Breeding Facility, Branch of the Shemyakin and Ovchinnikov Institute of Bioorganic Chemistry, Russian Academy of Sciences, (IBCh RAS Unique Research Device “Bio-model”, Pushchino, Russia). The mice were randomly divided into four groups: (1) non-treated control (CTR) (*n* = 9); (2) control + alisporivir (CTR+Ali) (*n* = 9); (3) DM (*n* = 9); and (4) mice with DM treated with alisporivir (DM+Ali) (*n* = 9). DM was induced in mice from the third and the fourth groups by means of high-fat diet feeding (Adjusted Calories Diet 60/Fat (ACD), Envigo, Indianapolis, IN, USA, #TD.06414) for 4 weeks (Figure 1A) [36]. This was followed by the daily administration of a low dose of streptozotocin (STZ) intraperitoneally (i.p) (30 mg/kg) for five consecutive days in continuation with ACD feeding. This model closely mimics the natural development of the DM as well as its metabolic features [37,38]. On the 33rd day, the ACD was withdrawn, and the mice were maintained on a normal balanced diet for four weeks. Control groups 1 and 2 were provided with a low-fat control diet (Envigo, Indianapolis, IN, USA, #TD.08806) and drinking water ad libitum, followed by vehicle, i.e., 0.1M citrate buffer (pH = 4.5) given i.p. On the 40th day, mice from groups 2 and 4 were treated with Ali (2.5 mg/kg/day, i.p.) for 20 days. Alisporivir (HY-12559, MedChemExpress, Monmouth Junction, NJ, USA) was dissolved in mixture of DMSO, ethanol, and sterile saline (12.5:25:62.5 *v/v*%). The sham-injected controls received solvent only. The successful induction of DM was confirmed by the intraperitoneal glucose tolerance test (IPGTT) and intraperitoneal insulin sensitivity test (IPIST). A stock solution of glucose (2 g/kg) in 0.1 mL of distilled water was administered i.p. to perform the IPGTT. To conduct the IPIST, human insulin was administrated at a dose of 1U/kg i.p. Before starting the tests, the mice were fasted for 16 h (IPGTT) and for 4 h (IPIST), with free access to water.

### 4.2. Electron Microscopy

Samples of the skeletal muscle (*M. quadriceps femoris*, two samples in each experimental group) were taken from a decapitated animal and were fixed for 2 h in a 2.5% glutaraldehyde solution in 0.1 M PBS (pH = 7.4) [39]. The preparations were examined and photographed using a JEM-100B electron microscope (JEOL, Tokyo, Japan). Ultrastructural analysis was performed using negative images digitized with an Epson V700 scanner. The morphometric analysis of the images was conducted on photographic negatives using the Image Tool 3.0 software.

### 4.3. RNA Extraction, Reverse Transcription, and Quantitative Real-Time PCR

Total RNA was isolated from 100 mg of deep-frozen tissue samples from the quadriceps using an ExtractRNA kit (#BC032, Eurogen, Moscow, Russia) in accordance with the protocols of the manufacturer. The real-time PCR was performed on a DTLite5 amplifier (DNA-Technology LLC, Moscow, Russia) using the qPCRmix-HS SYBR reaction mixture (Eurogen, Moscow, Russia). The selection and analysis of gene-specific primers were performed using Primer-BLAST [40] (the oligonucleotide sequences are presented in Table 2). The relative level of expression of each gene was normalized to the level of *Rplp2* mRNA, and a comparative C_T_ method was used to quantify the results [41].

### 4.4. Isolation of Skeletal Muscle Mitochondria

The mitochondria were isolated from the skeletal muscle tissue (quadriceps of both hindlimbs) by differential centrifugation as described earlier [39]. The final suspensions contained 30–40 mg of mitochondrial protein/mL. Quick Start Bradford Protein Assay (Bio-Rad Laboratories, Hercules, CA, USA) was used to quantify protein content.

### 4.5. Mitochondrial Respiration and Oxidative Phosphorylation

The rate of O_2_ consumption by the mitochondrial samples was estimated using Oxygraph-2k (Oroboros Instruments, Innsbruck, Austria) [42]. The reaction medium contained 120 mM KCl, 5 mM NaH_2_PO_4_, and 10 mM HEPES-KOH (pH = 7.4). The concentration of the mitochondrial protein in the cuvette was about 0.25 mg/mL.

### 4.6. Lipid Peroxidation

Lipid peroxidation in a suspension of isolated mitochondria was estimated spectrophotometrically by measuring the levels of thiobarbituric acid-reactive substances (TBARS). The TBARS assay quantifies the levels of malondialdehyde and other minor aldehyde species through their reaction with thiobarbituric acid [36].

### 4.7. Assay of the Mitochondrial Calcium Retention Capacity

The transport of Ca^2+^ across the inner mitochondrial membrane was monitored spectrophotometrically with an arsenazo III indicator at 675–685 nm using a Tecan Spark 10M plate reader (Tecan Group Ltd, Männedorf, Switzerland) as previously described [39]. Skeletal muscle mitochondria (~0.25 mg of mitochondrial protein/mL) were suspended in an incubation medium containing 150 mM sucrose, 50 mM KCl, 2 mM KH_2_PO_4_, 10 μM EGTA, and 10 mM HEPES-KOH (pH = 7.4) and were energized with 2.5 mM glutamate + 2.5 mM malate. To determine the ability of the mitochondria to retain Ca^2+^, 10 μM CaCl_2_ was added into the reaction medium successively, with an interval of about 90 s. After several additions, external (Ca^2+^) increased, indicating a massive release of the ion from the organelles due to the opening of the MPT pore in the inner mitochondrial membrane. The total amount of the added Ca^2+^ ions that induced their spontaneous release from mitochondria due to the induction of the MPT pore opening was interpreted as the calcium retention capacity of the organelles.

### 4.8. Electrophoresis and Immunoblotting of Mitochondrial OXPHOS Proteins

Total protein extracts were prepared from 10 mg of the frozen soleus muscle. To maintain extract integrity and function, Complete Protease Inhibitor Cocktail (P8340, Sigma-Aldrich, USA), Phosphatase Inhibitor Cocktail 3 (P0044 Sigma-Aldrich, USA), PMSF (1 mM), Na_3_VO_4_ (1 mM), and EGTA (1 mM), EDTA (1 mM) were used. Proteins were isolated using RIPA buffer (20–188, Millipore, MA, USA). Quick Start Bradford Protein Assay (Bio-Rad Laboratories, Hercules, CA, USA) was used to quantify protein content. The samples were diluted in Laemmli buffer, run on 12.5% SDS-PAGE (10 µg/lane), and transferred to a 0.45-µm nitrocellulose membrane (Amersham, Germany). Protein samples were denatured at 37 °C. The transfer and staining of the proteins were conducted according to Abcam protocols. The membranes were blocked with 2% non-fat milk (PBS) overnight at 4 °C. After blocking, the membranes were incubated with the appropriate primary antibody. The total OXPHOS Rodent WB Antibody Cocktail (ab110413) and the Anti-alpha Tubulin antibody (ab4074) were from Abcam (Cambridge, UK). As a positive control, rat heart tissue lysate–mitochondrial extract (contains SDS/DTT) (ab110341, Abcam, Cambridge, UK) was used (the letter *M* in Figure 4). The immunoreactivity was detected using the appropriate secondary antibody conjugated to horseradish peroxidase (7074 and 7076, Cell Signaling technology Inc., (Danvers, MA, USA). Peroxidase activity was detected with ECL chemiluminescence reagents (Pierce, Rockford, IL, USA). The relative levels of the detected proteins were visualized using a LI-COR system (LI-COR, Lincoln, NE, USA) and were normalized by the total protein concentration. Optical density measurements were performed by LI-COR Image Studio 5.2 software.

### 4.9. Statistical Analysis

The data were analyzed using the GraphPad Prism 7.0 software and were presented as mean ± SEM of 5–9 biological replicates (excluding electron microscopy data). The results of the transmission electron microscopy analysis were presented as representative images from two biological replicates. The statistical significance of the differences between the experimental groups was evaluated using one-way analysis of variance (ANOVA) followed by the Tukey multiple comparison post hoc test.

## 5. Conclusions

The data obtained suggest that alisporivir at the dose used is not a classic antidiabetic drug. Although there was a significant increase in the blood glucose clearance rate in the diabetic mice treated with alisporivir, the IPIST data show that the insulin sensitivity of these animals did not change significantly. One can assume that the use of a higher dose of alisporivir could have beneficial effects against the development of the pathology of diabetes mellitus.

At the same time, the administration of alisporivir at a dose of 2.5 mg/kg/day to diabetic mice (1) restores the morphology and cristae structure of mouse skeletal muscle mitochondria; (2) increases the expression of *Mfn2* and reduces the expression of *Drp1* genes encoding the proteins responsible for mitochondria fusion and fission, respectively; (3) recovers the respiratory control ratio and calcium retention capacity; (4) normalizes the susceptibility of skeletal muscle mitochondria to the MPT pore opening, and (5) reduces the intensity of lipid peroxidation. Altogether, this suggests that the use of alisporivir to target mitochondrial damage has the potential for a more comprehensive treatment for both causative and secondary defects in diabetes mellitus.

It is noteworthy that that the induction of diabetes mellitus is accompanied by a change in the levels of the main structural and regulatory proteins of the MPT pore in the skeletal muscles: a decrease in the expression of the genes encoding cyclophilin D, ATP synthase, and ANT1 and an increase in the expression level of the *Ant2* gene. This indicates that these proteins may be involved in the mechanisms that control the MPT pore formation in the skeletal muscle mitochondria of diabetic animals. These findings also suggest that the opening of the MPT pore is one of the triggers of mitochondrial damage in the skeletal muscles in diabetes mellitus, and targeting cyclophilin D and the MPT pore by alisporivir is a therapeutic approach to prevent the development of mitochondrial dysfunction and the associated oxidative stress in the skeletal muscles in diabetes.

## Figures and Tables

**Table 1:** Indicators of respiration and oxidative phosphorylation of the mouse skeletal muscle mitochondria of the experimental groups.

Group	V Respiration, nmol O_2_ × min^−1^ × mg^−1^ Protein				RCR	ADP/O
	State 2	State 3	State 4	State 3U_DNP_		
CTR	18.4 ± 0.7	162.9 ± 9.8	22.3 ± 1.2	196.3 ± 19.7	7.2 ± 0.1	2.98 ± 0.02
CTR+Ali	19.9 ± 0.6	174.8 ± 10.1	23.3 ± 1.0	225.4 ± 17.0	7.8 ± 0.5	2.85 ± 0.07
DM	18.0 ± 1.2	125.2 ± 4.8 *	25.1 ± 2.0	181.3 ± 20.5	5.1 ± 0.4 *	2.57 ± 0.13 *
DM+Ali	19.1 ± 1.1	158.7 ± 7.4 ^#^	24.8 ± 1.0	198.4 ± 20.2	6.6 ± 0.2 ^#^	2.77 ± 0.08

Incubation medium composition: 130 mM KCl, 5 mM NaH_2_PO_4_, 10 µM EGTA, and 10 mM HEPES-KOH, pH 7.4. Mitochondria respiration was fueled by 2.5 mM glutamate and 2.5 mM malate. State 3 respiration was initiated by 200 µM ADP. The results are presented as means ± SEM (*n* = 6). * *p* < 0.05 compared to the control group (CTR). ^#^ *p* < 0.05 compared to the DM group.

**Table 2:** List of gene-specific primers for RT-PCR analysis.

Gene	Forward (5′→3′)	Reverse (5′→3′)
*Atp5f1a*	GATCTATCCAAGCAGGCTGT	AGGCGGGAGTGTAGGTAGAA
*Ant1*	CTATGACACTGCCAAGGGGATG	TCAAACGGATAGGACACCAGC
*Ant2*	TCTGGACGCAAAGGAACTGA	GACCATGCGCCCTTGAAA
*Ppif*	GCAGATGTCGTGCCAAAGACTG	GCCATTGTGGTTGGTGAAGTCG
*D* *rp1*	TTACAGCACACAGGAATTGT	TTGTCACGGGCAACCTTTTA
*Mfn2*	CACGCTGATGCAGACGGAGAA	ATCCCAGCGGTTGTTCAGG
*Opa1*	GGACCCAAGAGCAGTGTGTT	CGAGACTCCAGGTTCTTCCG
*Ppargc1a*	CTGCCATTGTTAAGACCGAG	GTGTGAGGAGGGTCATCGTT
*Rplp2*	CGGCTCAACAAGGTCATCAGTGA	AGCAGAAACAGCCACAGCCCCAC

## Data Availability

The data presented in this study are available upon request from the corresponding author.
