# Correction: Belosludtsev et al. Alisporivir Treatment Alleviates Mitochondrial Dysfunction in the Skeletal Muscles of C57BL/6NCrl Mice with High-Fat Diet/Streptozotocin-Induced Diabetes Mellitus. *Int. J. Mol. Sci.* 2021, *22*, 9524

**DOI:** 10.3390/ijms252212080

**Published:** 2024-11-11

**Authors:** Konstantin N. Belosludtsev, Vlada S. Starinets, Eugeny Yu. Talanov, Irina B. Mikheeva, Mikhail V. Dubinin, Natalia V. Belosludtseva

**Affiliations:** 1 Department of Biochemistry, Cell Biology and Microbiology, Mari State University, pl. Lenina 1, 424001 Yoshkar-Ola, Russia; vlastar@list.ru (V.S.S.); dubinin1989@gmail.com (M.V.D.); 2 Laboratory of Mitochondrial Transport, Institute of Theoretical and Experimental Biophysics, Russian Academy of Sciences, Institutskaya 3, 142290 Pushchino, Russia; evg-talanov@yandex.ru (E.Y.T.); mikheirina@yandex.ru (I.B.M.); nata.imagination@gmail.com (N.V.B.)

The original publication [1] was lacking a reference to a previous study [2] conducted on the same cohort of mice and published by the same authors, from which panels (a), (c), and (d) of Figure 1 (the validation of a mouse model of diabetes using the intraperitoneal glucose tolerance test) was reused without citation. The correct legend for Figure 1 is shown below. 

The authors state that the scientific conclusions are unaffected. This correction was approved by the Academic Editor. The original publication has also been updated.

## Figures and Tables

**Figure 1:**
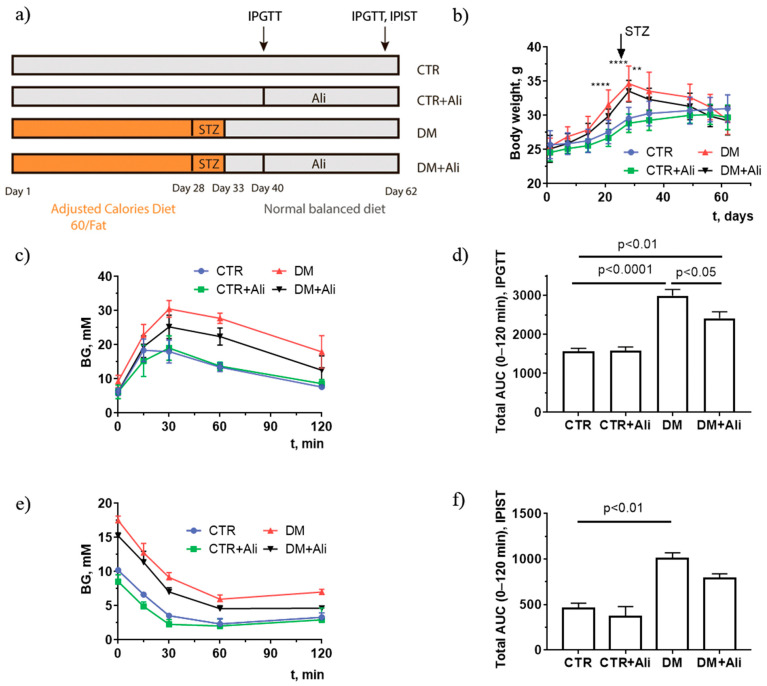
Induction scheme of diabetes mellitus (**a**), body weight gain (**b**), intraperitoneal glucose tolerance test, IPGTT (**c**), and intraperitoneal insulin sensitivity test, IPIST (**e**) in control (CTR), alisporivir-treated control (CTR+Ali), diabetic (DM), and alisporivir-treated diabetic (DM+Ali) mice. The total areas under the curve (AUC) of the IPGTT (**d**) and IPIST (**f**) are shown. The tests were conducted on the 60th day from the beginning of the experiment. ** In subfigure b, the difference between the CTR and DM+Ali groups is significant at *p* < 0.01. **** differences between the CTR and DM groups are significant at *p* < 0.0001. All data are presented as mean ± SEM (*n* = 5). Data in subfigures (**a**,**c**,**d**) are from Belosludtseva et al. *Biology* 2021, *10*, 839, doi: 10.3390/biology10090839.
